# Visual context due to speech-reading suppresses the auditory response to acoustic interruptions in speech

**DOI:** 10.3389/fnins.2014.00173

**Published:** 2014-07-02

**Authors:** Jyoti Bhat, Mark A. Pitt, Antoine J. Shahin

**Affiliations:** ^1^Department of Otolaryngology-Head and Neck Surgery, College of Medicine, The Ohio State UniversityColumbus, OH, USA; ^2^Department of Psychology, The Ohio State UniversityColumbus, OH, USA

**Keywords:** audiovisual integration, auditory evoked potentials, degraded speech, illusory filling-in, phonemic restoration

## Abstract

Speech reading enhances auditory perception in noise. One means by which this perceptual facilitation comes about is through information from visual networks reinforcing the encoding of the congruent speech signal by ignoring interfering acoustic signals. We tested this hypothesis neurophysiologically by acquiring EEG while individuals listened to words with a fixed portion of each word replaced by white noise. Congruent (meaningful) or incongruent (reversed frames) mouth movements accompanied the words. Individuals judged whether they heard the words as continuous (*illusion*) or interrupted (*illusion failure*) through the noise. We hypothesized that congruent, as opposed to incongruent, mouth movements should further enhance illusory perception by suppressing the auditory cortex's response to interruption onsets and offsets. Indeed, we found that the N1 auditory evoked potential (AEP) to noise onsets and offsets was reduced when individuals experienced the illusion during congruent, but not incongruent, audiovisual streams. This N1 inhibitory effect was most prominent at noise offsets, suggesting that visual influences on auditory perception are instigated to a greater extent during noisy periods. These findings suggest that visual context due to speech-reading disengages (inhibits) neural processes associated with interfering sounds (e.g., noisy interruptions) during speech perception.

## Introduction

The integration of auditory and visual information, such as combining speech-reading with listening, increases comprehension, especially in noisy conditions and in individuals with hearing loss (Sumby and Pollack, 1954; Grant and Seitz, 2000; Kaiser et al., 2003; Ross et al., 2007; Zion Golumbic et al., 2013). Basic research has provided insights into the neural functioning of audiovisual (AV) integration in speech processing. An emerging theory posits that AV integration is partly mediated via temporal alignment of the neural response to mouth movements with the response representing the contour of the speech envelope (Chandrasekaran and Ghazanfar, 2009; Luo et al., 2010). One test of this theory is to examine how it fares in adverse acoustic environments (Bishop and Miller, 2009; Shahin et al., 2009; Zion Golumbic et al., 2013). That is, how does the temporal coherence between mouth movements and the speech envelope affect the perception of degraded (noise-interrupted) speech? The theory predicts that a robust encoding of the speech signal, i.e., the contour of speech envelope, should be strengthened by simultaneously disengaging (inhibiting) neural processes associated with interfering sounds.

To this end, Shahin et al. (2012) tested this theory by examining the influence of visual context provided during speech-reading on illusory filling-in. Illusory filling-in occurs when individuals perceive a noise-interrupted sound as continuous through the noisy segment. Previous accounts (Riecke et al., 2009; Shahin et al., 2009, 2012) concluded that this phenomenon is partly accomplished by suppressing the auditory cortex (AC) response to the onsets/offsets of noisy interruptions, creating the illusion that the sound (speech) envelope is continuous. Shahin et al. (2012) hypothesized that visual context should further enhance this inhibitory process, thereby reinforcing illusory perception. They reasoned that this should be the case because a coupling of the heard speech with speech-reading enhances encoding of the speech envelope in AC (Zion Golumbic et al., 2013). In turn, the AC response to noisy signals that do not conform to the speech envelope should be inhibited to reduce AC sensitivity to interfering signals. To probe this premise, Shahin et al. (2012) examined auditory evoked potentials (AEPs) time-locked to the onsets and offsets of noise-interruptions while individuals listened to noise-interrupted words and judged whether they heard the words as continuous (experienced the illusion) or interrupted (failed to experience the illusion). The participants made these judgments while they watched mouth movements that were *congruent* (meaningful), *incongruent* (reversed frames), or *static* (no movements) with the spoken words.

Contrary to Shahin et al.'s (2012) prediction, *congruent* visual cues did not weaken AEPs to interruption boundaries (onsets and offsets) more so than *incongruent* or *static* mouth movements. However, individuals tolerated longer interruptions for the *congruent* vs. the *incongruent* and *static* conditions. This latter result was due to a key experimental design parameter, in which interruption duration was adaptively allowed to reach the maximum duration (ceiling) at which the participant could still perceive illusory continuity. Accordingly, one reason for the null effect in Shahin et al. (2012) is that variation in interruption duration might have masked AEP inhibitory evidence, especially in the *congruent* condition. That is, by allowing the noise duration to reach a participant's threshold (maximum), AEPs in all conditions may have reached the same maximum possible amplitude, creating a ceiling effect.

In the present study, we hypothesized that visually-induced AC inhibition might be observed by examining the AC response to interruption boundaries in which the interruption duration was equal between the *congruent* and *incongruent* conditions, but always below each participant's threshold. Thus, theoretically the interruption duration should be further below ceiling in the *congruent* than *incongruent* condition. Hence, we expect that, in both the *congruent* and *incongruent* conditions, the AC response to interruption boundaries to be smaller during illusory perception than when the illusion fails. However, this difference should occur to a greater extent in the *congruent* than *incongruent* condition, as Shahin et al. (2012) originally proposed. Finally, this neurophysiological effect should be reflected behaviorally as an increase in the number of trials labeled as *illusion* in the *congruent* than *incongruent* condition.

## Materials and methods

### Subjects

Fourteen native English speakers (average age = 27 years, range 18–60 years old; 8 females; 13 right handed and 1 left handed) with no known hearing problems participated in this study. All were between the ages of 18 and 30, except for one who was 60 years of age. The data from this individual and another participant were not included in the analyses because they had fewer than 12 valid trials in one condition of the EEG data. Handedness was determined using the Edinburgh Handedness Inventory. The study was conducted at the Auditory Neuroscience Lab, The Ohio State University Department of Otolaryngology and was approved by the local Institutional Review Board. The experiments were undertaken with the understanding and written consent of each subject, and the study conformed to The Code of Ethics of the World Medical Association (Declaration of Helsinki).

### Stimuli

**Figure 1:**
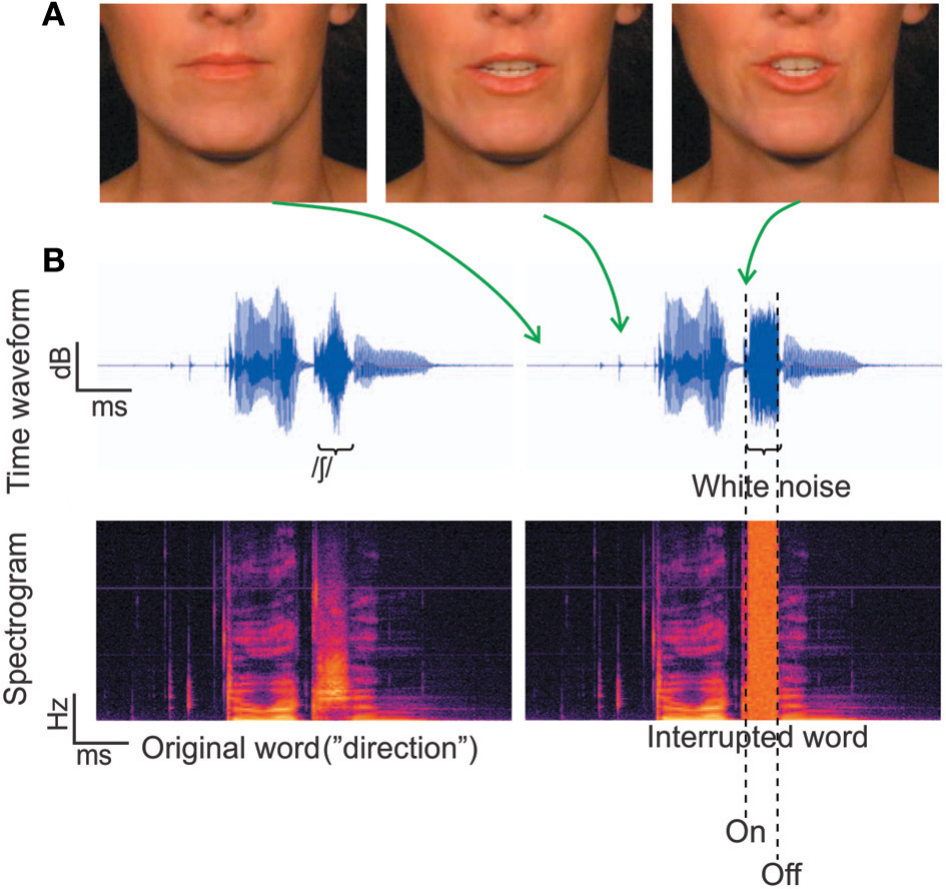
**Stimuli**. **(A)** Three frames corresponding to different time points along the utterance of the word “direction.” **(B)** The temporal waveforms and corresponding spectrograms for the word “direction.” The left panel depicts the original word with no noise, which was not used in the current experiment. The right panel depicts a physically interrupted word, where white noise replaced 100% of the fricative / ∫ /.

The auditory and visual stimuli were the same as those used in Shahin et al. (2012) (Figures 1A,B). Briefly, the stimuli consisted of 230 trisyllabic audiovisual words which were segregated into auditory (2550 ms) and visual (85 frames) segments. This ensured that the extracted frames and corresponding acoustic signal covered the entire mouth-movements and speech signal, respectively, with several frames with still (closed neutral position) lip-movements and silence at the beginning and end. There were three conditions: *static, congruent* and *incongruent*. In the *static* condition a still picture of the corresponding face accompanied auditory presentation of the word. In the *congruent* condition, mouth movements were synchronized with the acoustic word. In the *incongruent* condition, the frames of the *congruent* condition were reversed during word presentation, which was done to keep the visual motion at the same overall energy as the *congruent* condition. This is important to rule out physical differences in stimuli causing EEG effects.

### Procedure and task

#### Behavior

The experiment began with a behavioral (calibration) session using the *static* condition only, in which the maximum threshold of interruption duration resulting in perception of continuity was adaptively measured for each subject. Individuals sat in a sound attenuated room 1 meter in front of a 24 inch computer monitor and wore insert earphones (ER-4B Etymotic Research, Elk Grove Village, IL). Sound level was adjusted to the subject's comfort level (range 65 ± 5 dB) and kept constant throughout the experiment. Individuals listened to all 230 words randomly presented while fixating on a still face (*static*). All words were interrupted by white noise that was of the same intensity +3 dB SPL as the replaced segment. The duration of the replaced part of the word (i.e., white noise duration), which was always centered on a fricative or affricate ([t∫], [ʤ], [d], [s], [∫], [z]), was adjusted adaptively from trial to trial. We should note that the center of the replaced phoneme was 480 ± 124 ms following voice onset, placing it, on average, in the center of the word (words had average length of ~1 s). Also, the first mouth movement was 320 ± 144 ms prior to voice onsets. Because the noise-replaced phonemes varied in absolute duration, the duration of the noise itself was adjusted as a proportion of the phoneme's total duration. 100% of the fricative/affricative was replaced by white noise in the first trial of the adaptive procedure. If individuals responded that the word continued behind the noise (*illusion*), the white noise duration was increased by 15% (7.5% on each end of the noise) for the next trial. However, if individuals identified the word as interrupted (*illusion-failure*), noise duration was decreased by 15% for the next trial, and so forth. The calibration session lasted 15 min. Afterwards, the mean duration of white noise across trials and across the *illusion* and *illusion-failure* percepts for each individual was calculated. Following the calibration test, a fricative/affricate in all 230 words was replaced by white noise of the duration obtained from the calibration session for use in the EEG session. It is important to emphasize that the noise duration was fixed for an individual, and did not vary across the *congruent* and *incongruent* conditions.

#### EEG

**Figure 2:**
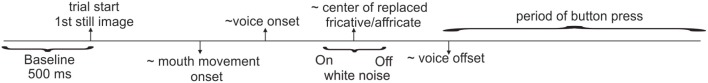
**Trial depiction**. Approximate timing of unfolding events during one trial.

Continuous EEG data were acquired using a BioSemi ActiveTwo system (Amsterdam, Netherlands; 64-channel cap, 10–20 system, Ag-AgCl electrodes, sampled at 512 Hz). The two passive electrodes Common Mode Sense (CMS) and the Driven Right Leg (DRL) served as ground. There were two blocks of interrupted word presentations. There were two additional blocks of control trials in which intact (non-interrupted) words were presented with the *congruent* and *incongruent* visual streams. These additional blocks were included to test other hypotheses unrelated to the current study. All blocks were randomized between subjects to rule out order effects. Each of the main blocks was 15 min long and contained all 230 noise-inserted words randomly presented. In one block, 115 interrupted words were presented with *congruent* visual stimuli and the other 115 words were presented with *incongruent* visual stimuli. In the second block, the *congruent* and *incongruent* word pairings were reversed. Thus, the only difference among these two conditions was the visual presentation (the acoustic stimuli, including the phonemes that were replaced and white noise durations, were identical). Block order was randomized across subjects. Each stimulus presentation was followed by a silent period of 1 s along with the still picture frame of the last displayed face. The average trial duration was set to 4.6 s. However, the inter-stimulus interval (ISI) of the spoken words was 2.55_*M*_ ± 0.32*_SD_* s, since sound onsets occurred at variable times in each trial. Figure 2 depicts the approximate timing of the unfolding events on a trial. Subjects pressed a button with their left index finger when they perceived the stimulus as continuous, and pressed another button with their left middle finger when they perceived it as interrupted. During the experiment, subjects were instructed to focus their attention on the talker's mouth and base their decision on the continuity (not the meaning) of the spoken word while ignoring the white noise.

### Data analysis

#### Behavior

The number of *illusion* and *illusion-failure* responses in the two congruency conditions (*congruent, incongruent*) was calculated for each participant.

#### EEG

Using EEGLAB (Delorme and Makeig, 2004) and in-house MATLAB code, continuous EEG files for all blocks were concatenated into one grand continuous file and filtered using a high pass Butterworth filter (>0.5 Hz). This file was then epoched into trials (regardless of condition type) from −500 to 4000 ms around trial onset (onset of 1st frame of the video). Trials were then average referenced, baselined to the 500 ms pre-stimulus period and corrected for ocular artifacts using independent component analysis (ICA). Then the trials were re-epoched from −200 to 1500 ms around the onsets of interruptions. Trials containing amplitudes of ±150 μV or greater in any channel were rejected. Data were then separated according to percept type (*illusion and illusion*-*failure*) and congruency condition (*congruent, incongruent*) and re-epoched around the onsets and offsets of interruptions between −200 to 500 ms. This re-epoching allowed us to examine electrophysiological changes between percepts and congruency conditions as a function of time-locking condition (onset or offset of interruption). There was no further baselining. Thus, the same baseline was maintained for both the onset and offset time-locking conditions. This was done to make sure that possible effects differentiating the two conditions were not attributed to different baseline periods. The mean number of trials in each condition that was included in the analysis was as follows: *congruent* − *illusion* = 148_*MN*_ ± 40*_SD_, congruent* − *illusion* − *failure* = 61_*MN*_ ± 33*_SD_, incongruent* − *illusion* = 113_*MN*_ ± 35*_SD_, incongruent* − *illusion* − *failure* = 90_*MN*_ ± 30*_SD_*. The overall number of *illusion* trials (~130) exceeded that of *illusion-failure* (~75) trials. This discrepancy is not unexpected and shows that individuals experienced the *illusion* more often than its failure. Finally, auditory evoked potentials (AEPs) were generated by averaging trials for each subject, channel, time-locking condition, congruency condition and percept type. The mean potential of each individual/condition was subtracted from the AEP averages. Data from two subjects were not included in the final EEG analysis because they had too few trials (<30 trials) in one of the conditions.

We limited our AEP analyses to the N1 and P2 AEPs since both of these AEPs exhibited changes with illusory perception in Shahin et al. (2012). We concentrated on two regions of interest (ROIs) where auditory activity is known to be dominant, the fronto-central region (channels F1, Fz, F2, FC1, FCz, FC2) and centro-parietal region (channels C1, Cz, C2, CP1, CP2, CP3). To obtain peak amplitudes we adopted a technique motivated by Clayson et al. (2013): 1) Peak latencies of the N1 and P2 AEPs were obtained from the group mean for each ROI and condition. 2) Individual N1 or P2 mean peak amplitudes (±10 ms) centered on the group latency values in step (1) were obtained for each ROI and condition. This method led to the following windows being used. For the N1 AEP at centro-parietal ROI, the window of analysis was consistent across conditions due to the consistent latency of the N1 peak: we used a window of 90–110 ms for the *congruent illusion* and *illusion-failure* percept types at the onset and offset; we used a window of 90–110 ms for the *incongruent illusion-failure* percept at onset and offset, and we used a window of 86–106 ms for the *incongruent illusion* percept at onset and offset. For the P2 AEP at centro-parietal ROI, the window of analyses exhibited high variability between conditions: this resulted in using a window of 165–185 ms for the *congruent* and incongruent *illusion-failure* percept at the onset; we used a window of 180–200 ms for the *congruent illusion-failure* percept at the offset, and a window of 195–215 ms for the *incongruent illusion-failure* percept at the offset; we used a window of 205–225 ms for the *congruent illusion* percept at the onset, a window of 140–160 ms for the *incongruent illusion* percept at the onset, a window of 210–230 ms for the *congruent illusion* percept at the offset, and a window of 175–195 ms for the *incongruent illusion* percept at the offset. Similar values were obtained for the fronto-central ROI.

### Statistical analyses

#### Behavior

For each subject, we first calculated the percentage of *illusion* and *illusion-failure* responses in each congruency condition. We then normalized the percept classifications (number of responses of *illusion* or *illusion-failure*) to percentages of the overall response within a congruency condition. Then we conducted repeated measures analysis of variance (ANOVA) contrasting classification percentages across conditions with the independent variables being congruency (*congruent, incongruent*) × percept type (*illusion, illusion-failure*). *Post-hoc* analyses were based on the Newman-Keuls test.

#### EEG

We conducted separate ANOVAs for the N1 and P2 AEPs (amplitude or latency), with the independent variables being percept type × time-locking × congruency. *Post hoc* analyses were performed using the Newman-Keuls test.

## Results

### Behavior

Inspection of the data from the calibration session (prior to EEG recording) showed that the duration of the noise interruption exceeded the fricative duration. The group average threshold for the perception of continuity (*illusion*) was 187_*MN*_ ± 64*_SD_*% of the average duration of the replaced phoneme, which translates to a group average duration of 281_*MN*_ ± 96*_SD_* ms. This result shows that perception of the *illusion* was not confined to the phoneme on which the noise interruption was centered, but also extended to adjacent phonemes.

**Figure 3:**
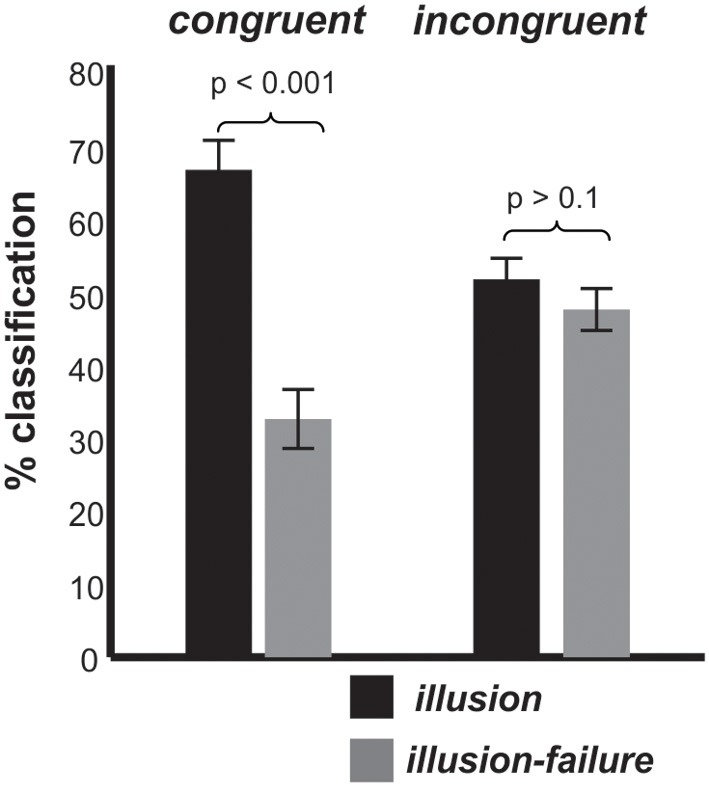
**Group mean classification (*illusion, illusion-failure*) percentages and standard deviations for the *congruent* and *incongruent* conditions;** *illusion* implies that the individuals classified the interrupted stimuli as continuous, while *illusion-failure* implies that the individuals classified the interrupted stimuli as interrupted.

Turning to the behavioral data collected during the EEG session, Figure 3 shows how often individuals classified the stimuli as interrupted (*illusion-failure*) or continuous (*illusion*) for the *congruent* and *incongruent* conditions. Recall that the physical attributes of auditory stimuli were identical in the two congruency conditions. Thus, any difference in classification must be due to a difference in perception, and cannot be due to physical differences in the stimuli. An ANOVA with variables congruency and percept type revealed a main effect of percept type [*F*_(1, 11)_ = 9.6, *p* < 0.01, η*_p_* = 0.46] and an interaction between percept type and congruency [*F*_(1, 11)_ = 21.0, *p* < 0.005, η*_p_* = 0.65]. The main effect of percept type was attributed to a greater number of trials labeled as continuous (*illusion*) than interrupted (*illusion-failure*). The interaction between the variables was attributed to a stronger *illusion* (a greater number of trials labeled as continuous vs. interrupted) occurring in the *congruent* than *incongruent* condition *(congruent: p < 0.001; incongruent > 0.1;* Newman-Keuls).

### EEG

**Figure 4:**
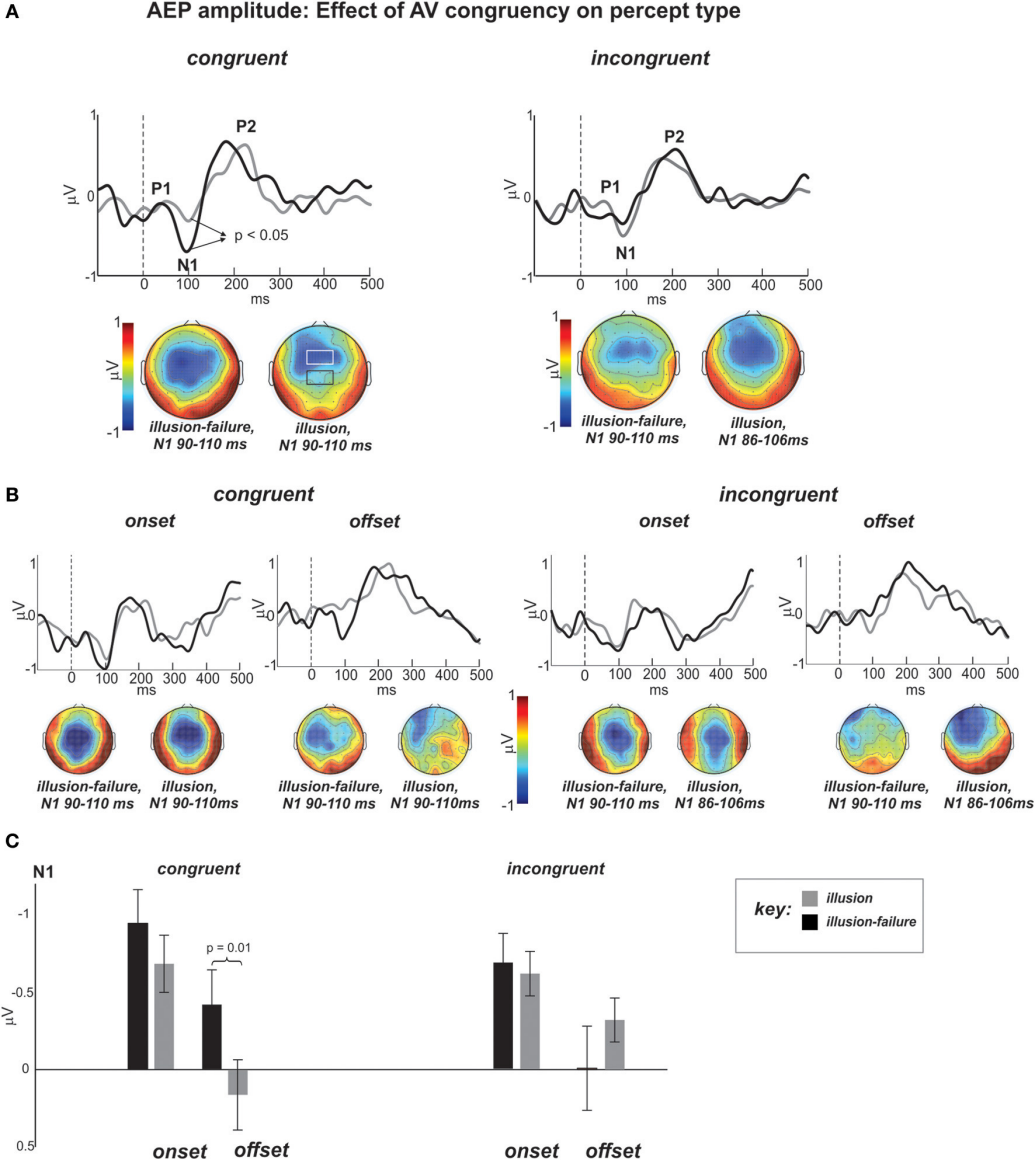
**(A)** Group average AEP waveforms of the *illusion* (gray) and *illusion-failure* (black) percepts (superimposed) for the *congruent* and *incongruent* conditions averaged across onsets and offsets of interruptions. The waveforms represent the average across the centro-parietal channels (C1, C2, Cz, CP1, CP2, CPz, see black-outlined box of the middle N1 topography of the congruent condition). The topographies at the bottom represent the mean potential distribution over a 20 ms window around the N1 peak. Dashed lines at 0 ms represent sound interruption onset and offset. **(B)** AEP waveforms and topographies of the N1 separated according to the onset and offset of interruptions time-locking conditions.**(C)** Bar graph depicting the mean N1 amplitudes and standard errors for all conditions.

As a reminder, our hypothesis stated that the weakening of N1-P2 AEPs to onsets and offsets of noise-interruptions during *illusion* vs. *illusion-failure* perception should be greater in the *congruent* than *incongruent* streams. Figure 4A shows the group average AEP waveforms of the *illusion* and *illusion-failure* percepts (superimposed) in the *congruent* and *incongruent* conditions (averaging across onsets and offsets of interruptions). Figure 4B shows the waveforms when the data were further segregated according to onset and offset conditions. They represent the average waveforms across channels in the centro-parietal ROI shown in the black-outlined box of the middle N1 topography of the *congruent* condition. The channels comprising the fronto-central ROI are shown in the more anterior white-lined box. Below the waveforms are N1 topographies (mean topographies of 20 ms around the peak) for the two percept types. In line with our predictions, notice, that in the waveforms of the *congruent* condition the N1 is more prominent for the *illusion-failure* (black waveforms) than the *illusion* (gray waveforms) percept. This relationship is not realized in the *incongruent* condition. These observations were tested statistically.

#### AEP amplitude

We first conducted *t*-tests comparing fronto-central and centro-parietal topographies (collapsing across all conditions). The *t*-tests approached significance for the N1 AEP (*p* = 0.06) and was highly significant for the P2 AEP (*p* = 0.0001). These effects were due to greater N1 and P2 amplitudes observed at fronto-central ROI than centro-parietal ROI. These results warranted separate ANOVAs for the fronto-central and centro-parietal ROIs.

Also, statistical analyses of the data at the fronto-central ROI revealed only a main effect of boundary for the N1 and P2 AEPs (*p* < 0.05). The N1 and P2 AEP amplitudes were larger following the onsets than offsets of interruptions. Because there were no effects of the main variables of interest (e.g., congruency), we turned our attention to the centro-parietal region, where AEP amplitude differences reflected illusory perception and congruency effects.

#### N1 AEP amplitude

Figure 4C shows the bar graphs summarizing the ANOVA contrasting the N1 amplitude across conditions at the centro-parietal ROI. An ANOVA on the N1 AEP, with variables percept type, time-locking (onsets and offsets), and congruency, revealed a main effects of percept type approaching significance [*F*_(1, 11)_ = 4.1, *p* < 0.07, η*_p_* = 0.27], which was due to smaller N1 AEPs occurring for the *illusion* than the *illusion-failure* percept across *congruency and time-locking* conditions. This result is in line with the findings of Shahin et al. (2012). There was also an interaction between the variables percept type and congruency [*F*_(1, 11)_ = 8.25, *p* < 0.02, η*_p_* = 0.42]. *Post hoc* tests revealed that this effect was due to smaller N1 amplitudes occurring for the *illusion* than *illusion-failure* percepts only in the *congruent*, not *incongruent*, condition (Newman-Keuls, *p* < 0.05, Figure 4C). However, there also was an interaction among all three variables that further differentiated the N1 effect [*F*_(1, 11)_ = 5.0, *p* < 0.05, η*_p_* = 0.31]. *Post hoc* tests revealed that the N1 suppression distinguishing *illusion* from *illusion-failure* percepts in the *congruent* condition was greater at the offsets than onsets of interruptions (Newman-Keuls, *p* < 0.02, Figure 4C). In the *incongruent* condition, not only was the difference not reliable, but it was in the opposite direction.

#### P2 AEP amplitude

An ANOVA on the P2 amplitude data revealed only a main effect of percept type [*F*_(1, 11)_ = 8.25, *p* < 0.02, η*_p_* = 0.42], which was attributed to smaller P2 amplitudes occurring for the *illusion* than *illusion-failure* percepts (mean and standard error of *illusion-failure* 0.59 ± 0.15; mean and standard error of *illusion* 0.44 ± 0.16). This result is also consistent with the premise that weakening of AEPs is consistent with illusory perception (Shahin et al., 2012).

***Summary of AEP amplitude results.*** In sum, the N1 amplitude results support the premise that the neurophysiological basis for illusory perception—suppression of the AC response to interruption boundaries during continuity perception—was only observed when the speech was accompanied by meaningful speech-reading (*congruent* visual streams). This effect was localized to the centro-parietal portion of the scalp. In contrast, the P2 AEP was not influenced by visual context, although, like the N1 AEP, its inhibition for the *illusion* vs. *illusion-failure* percepts indexed continuity perception.

#### AEP latency

Analyses of N1 and P2 latencies yielded no differences as a function of congruency, but yielded differences between percept types and time-locking conditions. Because the fronto-central and centro-parietal ROIs yielded qualitatively similar results, we report only the latency effects of the centro-parietal ROI.

#### N1 AEP latency

An ANOVA on the N1 latency data revealed only a main effect of time-locking [*F*_(1, 11)_ = 6.7, *p* < 0.05, η*_p_* = 0.37], which was due to shorter latencies occurring at the offsets than onsets of interruptions.

#### P2 AEP latency

An ANOVA on the P2 latency data revealed a main effect of percept type [*F*_(1, 11)_ = 181, *p* < 0.0001, η*_p_* = 0.99] and a main effect of time-locking [*F*_(1, 11)_ = 154, *p* < 0.0001, η*_p_* = 0.93]. There was also an interaction between percept type and time-locking [*F*_(1, 11)_ = 18, *p* < 0.005, η*_p_* = 0.62]. Shorter latencies occurred for the *illusion* than *illusion-failure* percepts, but this difference was greater for the onsets than offsets of interruptions (*p* < 0.005).

## Discussion

Our study demonstrates that visual information provided by speech-reading reinforces inhibition of the AC response to noisy interruptions, thus increasing perceptual tolerance for degraded speech. The N1 results—suppression at interruption boundaries during illusory perception compared to when the illusion failed—replicate those of Shahin et al. (2012). However, this study reports a new finding: N1 inhibition is present during speech-reading of *congruent* but not *incongruent* audiovisual speech streams. This neurophysiological effect was also reflected behaviorally, whereby individuals classified the interrupted words as continuous (experienced the illusion) more often during the *congruent* than *incongruent* AV streams. A logical next question to ask is: *What are the neural dynamics that facilitate visual enhancement of illusory filling-in?* Because we used EEG in this study, it is not feasible to identify all brain regions involved during the AV task. However, by integrating the current findings with a synthesis of the neural dynamics described in previous research, a plausible account can be offered.

It has been well established that the N1 AEP represents neural activity generated in the primary auditory cortex (PAC) and surrounding areas, such as the belt region of non-PAC (Scherg and Von Cramon, 1985; Pantev et al., 1995; Picton et al., 1999). Thus, the N1 suppressive effect can be explained as a decrease of neural recruitment and/or temporal alignment of neuronal firings in PAC to stimulus boundaries. Therefore, we can conclude that this inhibitory process weakens this region's sensitivity to interruption onsets and offsets that do not conform to the fidelity (smoothness) of the speech envelope. This in turn heightens the perception of speech continuity through the noise. This position is consistent with earlier conclusions from EEG and fMRI studies on illusory continuity (Heinrich et al., 2008; Riecke et al., 2009; Shahin et al., 2009), which reported that greater tolerance for degraded speech (enhanced continuity perception) co-occurs with decreased activity at PAC.

Missing from the preceding account is an explanation of the relevance of visual information to the observed suppressive N1 effect during perception of degraded speech. Visually-mediated suppression of the N1 AEP is not without precedence. It has been well reported in prior studies, using non-noisy speech, that visual influence on AEPs is suppressive (Besle et al., 2004; van Wassenhove et al., 2005; Stekelenburg and Vroomen, 2007). However, at the same time there is strong evidence suggesting that vision recruits higher-level networks along the auditory system, i.e., non-PAC, during visual integration. The classical view posits that AV integration occurs via the posterior superior temporal sulcus-gyrus (pSTS-G) and associated inter-sensory regions such as the middle temporal gyrus (MTG) and intra-parietal sulcus (IPS) (Calvert and Campbell, 2003; Beauchamp et al., 2004a,b; Miller and D'Esposito, 2005). These previous accounts, taken in combination with ours, lead us to posit that visually-mediated suppression of the N1 AEP may be part of an ascending shift of activity along the auditory system, primed by vision. More specifically, visual context predicts the unfolding cues in the speech signal (e.g., those revealing phonetic identity such as rhythm or formant transition) and causes auditory processing to be reweighted (re-routed) from low-level auditory networks (e.g., PAC, N1 inhibition) to high-level ones (non-PAC, excitation).

The N1 suppressive effect may be related to the audiovisual system's ability to reduce phase resetting of ongoing AC oscillatory activity in the alpha or theta bands (Hanslmayr et al., 2007; Fuentemilla et al., 2009) along acoustic boundaries (Luo and Poeppel, 2007). This reduction in phase-resetting may enhance tracking of the speech envelope (Luo and Poeppel, 2007) through the interruptions, and hence augment illusory continuity. This premise supports the findings of Shahin et al. (2012), who found that a reduction in N1 amplitude at the onsets and offsets of interruptions was accompanied by reduced phase-resetting of theta band.

This reweighting hypothesis fits within the auditory system's objective to efficiently organize incoming speech representations along the auditory system, such that contextual information prevails over transient spectrotemporal cues to ensure object recognition. Indeed, both animal and human neuroimaging studies have concluded that simple sounds are favorably processed at PAC; however as sounds become more meaningful (e.g., more structured and familiar), processing shifts to non-PAC (e.g., superior temporal sulcus/gyrus, middle temporal gyrus), and even higher-level areas (e.g., fronto-parietal and motor regions) (Rauschecker et al., 1995; Hickok and Poeppel, 2000, 2007; Tian et al., 2001; Wessinger et al., 2001; Patterson et al., 2002; Pasley et al., 2012). In light of these facts, we posit that vision must tap into these processes and reinforce the reweighting along the auditory system, allowing for complex auditory representations to be fused with visual representations. This is key to enhancing intelligibility across adverse acoustical situations.

To put the above reasoning into the context of the current study, the visually primed inhibition at PAC commences immediately following the onset of mouth movements (prior to the onset of speech or interruption). By the time the noisy interruption unfolds, the brain had already become less sensitive to simple features in sound, dampening the perceptual system's sensitivity to the onsets and offsets of interruptions. Supporting this premise is a study which reported that the observed N1 AEP suppression only occurred when visual anticipatory motion preceded the sound (Stekelenburg and Vroomen, 2007). In other words, the N1 effect was achieved only when visual cues were contextually relevant to the auditory cues, consistent with our results of greater N1 suppression during the *congruent* vs. *incongruent* conditions. However, at the same time the inhibitory process commences at PAC, vision excites higher level auditory networks, so contextual knowledge (phonological/lexical) can be engaged to aid filling-in of missing phonemic representations. In other words, the low-level inhibitory and high-level excitatory mechanisms (the reweighting) work in tandem to fulfill illusory perception. This is in line with earlier studies on illusory filling-in which reported that this process is driven by higher-level neural networks in the superior temporal sulcus, angular gyrus, middle temporal gyrus and inferior frontal gyrus (Heinrich et al., 2008, 2011; Shahin et al., 2009), while simultaneously activity at PAC is weakened. It may be that visual context primes those regions (PAC as well as the higher-level ones), reinforcing illusory filling-in.

We further posit that this visual influence is reinforced during adverse acoustic situations, in which phonetic and lexical information are not as clear as in quiet situations. This assessment is based on the finding that N1 suppression was most pronounced at the offsets, as opposed to the onsets, of interruptions. Our reasoning is that the onset of noise triggered greater reliance on visual modulation leading to increased tolerance for the unfolding noise, evidenced by greater suppression at AC at noise offset. This process may arise because of the growing necessity to encode the unfolding patterns of the speech envelop through visual modulation in noisy situations. A recent study of visual influence on auditory speech stream segregation (i.e., cocktail party Zion Golumbic et al., 2013) supports this conclusion. The authors reported that speech envelope tracking of the visually-attended stream in the AC was stronger when mouth movements were absent (auditory-only stream).

A caveat of the current experimental design relates to the N1 inhibitory results at the offset of interruptions distinguishing *congruent* and *incongruent* AV conditions. By temporally-locking AEPs to the offsets of interruptions we risked an overlap from preceding onset AEPs. Because the onsets and offsets of interruptions were separated on average by 281ms, the onset of P2 may have overlapped with the offset of N1. However, if we take the above group average values as a representative, the peaks of the onset P2 (~190 ms temporally-locked to onset of interruptions) and offset N1 (~ 100 ms + 281 ms temporally-locked to onset of interruptions) would still be separated by about 190 ms. Thus, an overlap would be most likely between the leading tail of the P2-onset with the lagging tail of the N1-offset. While this overlap effect may be small given that the window of analysis was ±10 ms around the peak, nonetheless we caution that the N1-offset results could have been modulated by P2 onset.

Our behavioral data are consistent with the view that visual context mitigates the disruptive effects of interruptions. Individuals perceived continuity for a larger number of words in the *congruent* than *incongruent* condition, suggesting that the visual context, when *congruent*, served to facilitate illusory-perception by reinforcing information common to both the ears and eyes. However, that illusory filling-in failed on congruent trials some of the time begs for an explanation. It is likely that some words contain phonemes or phonetic cues that are not as receptive to visual modulation as others. For example, the voiced/voiceless distinction is not conveyed visually, whereas place of articulation (labial vs. alveolar) is highly visible. These visually unreceptive cues are not limited to the fricatives/affricates originally replaced because the interruption covered on average 190% of the duration of the phoneme, and thus extended to cover, in part or whole, adjacent phonemes. Moreover, because mouth movements naturally lead the unfolding speech, the types of phonemes that preceded the interrupted phoneme likely played a role in mediating visual influence. Neurophysiologically, this may explain why the visually-induced inhibitory effect on the N1 AEP was only observed during a successful *illusion*, not when the *illusion* failed (i.e., the visual context of the *illusion-failure* percepts were unhelpful for both the *congruent* and *incongruent* conditions).

One outstanding question relates to the topographical differences in N1 AEP (Figure 4A). In the *congruent* condition, the *illusion-failure's* N1 is maximally exhibited at the center of the scalp, whereas the *illusion's* N1 is more frontally located. The difference of these two N1 AEPs resulted in a central topography (maximum) at Cz. The auditory N1 is known to span centro-frontal regions and several generators in PAC and surrounding areas. By subtracting the N1 AEPs of the *illusion* and *illusion-failure* percepts, we may have identified a region of the auditory cortex that corresponds to acoustic onsets and offsets. In a similar experimental design (Shahin et al., 2012), this region was localized to the middle portion of Heschl's Gyrus (PAC) using fMRI. Being localized to PAC as opposed to non-PAC is consistent with the earlier latency observed for the subtracted N1 (80 ms), hence reflecting earlier processes along the auditory pathway (Figure 4A). We should note that the mismatch of the difference topographies of the *congruent* and *incongruent* conditions suggests that different auditory generators underlie the *congruent* and *incongruent* effects.

In conclusion, our findings support the hypothesis that visual context via speech-reading weakens representations of interfering (non-conforming) signals (noisy interruptions). This could be due to a shift in processing toward high level auditory networks to take advantage of more complex acoustic features in speech. Our N1 result, along with prior research, begins to elucidate the neural mechanisms of AV integration of degraded speech and suggests avenues for further investigations. Namely the hypothesis can benefit from further investigations that manipulate the phonemic clarity of the visual information (e.g., sensitivity of replaced phoneme or preceding phonemes to visual influence) while simultaneously probing the behavior of low and high level auditory networks.

### Conflict of interest statement

The authors declare that the research was conducted in the absence of any commercial or financial relationships that could be construed as a potential conflict of interest.

## References

[B1] Beauchamp M. S. Argall B. D. Bodurka J. Duyn J. H. Martin A. (2004a). Unraveling multisensory integration: patchy organization within human STS multisensory cortex. Nat. Neurosci. 7, 1190–1192 10.1038/nn1333 15475952

[B2] Beauchamp M. S. Lee K. E. Argall B. D. Martin A. (2004b). Integration of auditory and visual information about objects in superior temporal sulcus. Neuron 41, 809–823 10.1016/S0896-6273(04)00070-4 15003179

[B3] Besle J. Fort A. Delpuech C. Giard M. H. (2004). Bimodal speech: early suppressive visual effects in human auditory cortex. Eur. J. Neurosci. 20, 2225–2234 10.1111/j.1460-9568.2004.03670.x 15450102 PMC1885424

[B4] Bishop C. W. Miller L. M. (2009). A multisensory cortical network for understanding speech in noise. J. Cogn. Neurosci. 21, 1790–1805 10.1162/jocn.2009.21118 18823249 PMC2833290

[B5] Calvert G. A. Campbell R. (2003). Reading speech from still and moving faces: the neural substrates of visible speech. J. Cogn. Neurosci. 15, 57–70 10.1162/089892903321107828 12590843

[B6] Chandrasekaran C. Ghazanfar A. A. (2009). Different neural frequency bands integrate faces and voices differently in the superior temporal sulcus. J. Neurophysiol. 101, 773–788 10.1152/jn.90843.2008 19036867 PMC2657063

[B7] Clayson P. E. Baldwin S. A. Larson M. J. (2013). How does noise affect amplitude and latency measurement of event-related potentials (ERPs)? A methodological critique and simulation study. Psychophysiology 50, 174–186 10.1111/psyp.12001 23216521

[B8] Delorme A. Makeig S. (2004). EEGLAB: an open source toolbox for analysis of single-trial EEG dynamics including independent component analysis. J. Neurosci. Methods 134, 9–21 10.1016/j.jneumeth.2003.10.009 15102499

[B9] Fuentemilla L. Marco-Pallarés J. Gual A. Escera C. Polo M. Grau C. (2009). Impaired theta phase-resetting underlying auditory N1 suppression in chronic alcoholism. Neuroreport 20, 337–342 10.1097/WNR.0b013e32832326ed 19188856

[B10] Grant K. W. Seitz P. F. (2000). The use of visible speech cues for improving auditory detection of spoken sentences. J. Acoust. Soc. Am. 108, 1197–1208 10.1121/1.1288668 11008820

[B11] Hanslmayr S. Klimesch W. Sauseng P. Gruber W. Doppelmayr M. Freunberger R. (2007). Alpha phase reset contributes to the generation of ERPs. Cereb. Cortex 17, 1–8 10.1093/cercor/bhj129 16452640

[B12] Heinrich A. Carlyon R. P. Davis M. H. Johnsrude I. S. (2008). Illusory vowels resulting from perceptual continuity: a functional magnetic resonance imaging study. J. Cogn. Neurosci. 20, 1737–1752 10.1162/jocn.2008.20069 18211243

[B13] Heinrich A. Carlyon R. P. Davis M. H. Johnsrude I. S. (2011). The continuity illusion does not depend on attentional state: FMRI evidence from illusory vowels. J. Cogn. Neurosci. 23, 2675–2689 10.1162/jocn.2011.21627 21268669

[B14] Hickok G. Poeppel D. (2000). Towards a functional neuroanatomy of speech perception. Trends Cogn. Sci. 4, 131–138 10.1016/S1364-6613(00)01463-7 10740277

[B15] Hickok G. Poeppel D. (2007). The cortical organization of speech processing. Nat. Rev. Neurosci. 8, 393–402 10.1038/nrn2113 17431404

[B16] Kaiser A. R. Kirk K. I. Lachs L. Pisoni D. B. (2003). Talker and lexical effects on audiovisual word recognition by adults with cochlear implants. J. Speech Lang. Hear. Res. 46, 390–404 10.1044/1092-4388(2003/032) 14700380 PMC3432920

[B17] Luo H. Liu Z. Poeppel D. (2010). Auditory cortex tracks both auditory and visual stimulus dynamics using low-frequency neuronal phase modulation. PLoS Biol. 8:e1000445 10.1371/journal.pbio.1000445 20711473 PMC2919416

[B18] Luo H. Poeppel D. (2007). Phase patterns of neuronal responses reliably discriminate speech in human auditory cortex. Neuron 54, 1001–1010 10.1016/j.neuron.2007.06.004 17582338 PMC2703451

[B19] Miller L. M. D'Esposito M. (2005). Perceptual fusion and stimulus coincidence in the cross-modal integration of speech. J. Neurosci. 25, 5884–5893 10.1523/JNEUROSCI.0896-05.2005 15976077 PMC6724802

[B20] Pantev C. Bertrand O. Eulitz C. Verkindt C. Hampson S. Schuierer G. (1995). Specific tonotopic organizations of different areas of the human auditory cortex revealed by simultaneous magnetic and electric recordings. Electroencephalogr. Clin. Neurophysiol. 94, 26–40 10.1016/0013-4694(94)00209-4 7530637

[B21] Pasley B. N. David S. V. Mesgarani N. Flinker A. Shamma S. A. Crone N. E. (2012). Reconstructing speech from human auditory cortex. PLoS Biol. 10:e1001251 10.1371/journal.pbio.1001251 22303281 PMC3269422

[B22] Patterson R. D. Uppenkamp S. Johnsrude I. S. Griffiths T. D. (2002). The processing of temporal pitch and melody information in auditory cortex. Neuron 36, 767–776 10.1016/S0896-6273(02)01060-7 12441063

[B23] Picton T. W. Alain C. Woods D. L. John M. S. Scherg M. Valdes-Sosa P. (1999). Intracerebral sources of human auditory-evoked potentials. Audiol. Neurootol. 4, 64–79 10.1159/000013823 9892757

[B24] Rauschecker J. P. Tian B. Hauser M. (1995). Processing of complex sounds in the macaque nonprimary auditory cortex. Science 268, 111–114 10.1126/science.7701330 7701330

[B25] Riecke L. Esposito F. Bonte M. Formisano E. (2009). Hearing illusory sounds in noise: the timing of sensory-perceptual transformations in auditory cortex. Neuron 64, 550–561 10.1016/j.neuron.2009.10.016 19945396

[B26] Ross L. A. Saint-Amour D. Leavitt V. M. Javitt D. C. Foxe J. J. (2007). Do you see what I am saying? Exploring visual enhancement of speech comprehension in noisy environments. Cereb. Cortex 17, 1147–1153 10.1093/cercor/bhl024 16785256

[B27] Scherg M. Von Cramon D. (1985). Two bilateral sources of the late AEP as identified by a spatio-temporal dipole model. Electroencephalogr. Clin. Neurophysiol. 62, 32–44 10.1016/0168-5597(85)90033-4 2578376

[B28] Shahin A. J. Bishop C. W. Miller L. M. (2009). Neural mechanisms for illusory filling-in of degraded speech. Neuroimage 44, 1133–1143 10.1016/j.neuroimage.2008.09.045 18977448 PMC2653101

[B29] Shahin A. J. Kerlin J. R. Bhat J. Miller L. M. (2012). Neural restoration of degraded audiovisual speech. Neuroimage 60, 530–538 10.1016/j.neuroimage.2011.11.097 22178454 PMC3288427

[B30] Stekelenburg J. J. Vroomen J. (2007). Neural correlates of multisensory integration of ecologically valid audiovisual events. J. Cogn. Neurosci. 19, 1964–1973 10.1162/jocn.2007.19.12.1964 17892381

[B31] Sumby W. H. Pollack I. (1954). Visual contribution to speech intelligibility in noise. J. Acoust. Soc. Am. 26, 212–215 10.1121/1.1907309

[B32] Tian B. Reser D. Durham A. Kustov A. Rauschecker J. P. (2001). Functional specialization in rhesus monkey auditory cortex. Science 292, 290–293 10.1126/science.1058911 11303104

[B33] van Wassenhove V. Grant K. W. Poeppel D. (2005). Visual speech speeds up the neural processing of auditory speech. Proc. Natl. Acad. Sci. USA. 102, 1181–1186 10.1073/pnas.0408949102 15647358 PMC545853

[B34] Wessinger C. M. VanMeter J. Tian B. Van Lare J. Pekar J. Rauschecker J. P. (2001). Hierarchical organization of the human auditory cortex revealed by functional magnetic resonance imaging. J. Cogn. Neurosci. 13, 1–7 10.1162/089892901564108 11224904

[B35] Zion Golumbic E. Cogan G. B. Schroeder C. E. Poeppel D. (2013). Visual input enhances selective speech envelope tracking in auditory cortex at a “cocktail party.” J. Neurosci. 33, 1417–1426 10.1523/JNEUROSCI.3675-12.2013 23345218 PMC3711546

